# The relationship between working memory updating components and reading comprehension

**DOI:** 10.1007/s10339-023-01127-3

**Published:** 2023-02-11

**Authors:** Rocío Linares, Santiago Pelegrina

**Affiliations:** Department of Psychology, University of Jaén, Paraje Las Lagunillas S/N, 23071 Jaén, Spain

**Keywords:** Reading comprehension, Working memory updating, Retrieval, Substitution, Working memory

## Abstract

The objective of this study was to determine the contribution of retrieval and substitution components of working memory updating to reading comprehension. Difficulties in reading comprehension have been related to the inability to update information in working memory. Updating is a complex process comprising various subprocesses, such as retrieving information into the focus of attention and substituting information that is no longer relevant. Various numerical subtasks requiring or not requiring the substitution and retrieval components of working memory updating, as well as reading comprehension and general cognitive measures, were administered to a sample (*n* = 148) of 4th grade children. Less-skilled comprehenders showed lower accuracy when information retrieval was required. In contrast, substitution was not related to reading comprehension. These findings suggest that reading comprehension difficulties are related to the efficacy of information retrieval during updating in working memory.

**Supplementary Information:**

The online version contains supplementary material available at 10.1007/s10339-023-01127-3.

## Introduction

Reading comprehension (RC) is one of the most important abilities for various human activities (Kendeou et al. 2016) and requires the complex integration of multiple general language skills (Nouwens et al. 2016) and cognitive processes (Muijseelar and De Jong 2015), such as working memory (WM).

WM is a capacity-limited system that maintains and processes selected representations required for performing numerous cognitive tasks. WM capacity determines an individual’s ability to integrate stored text representations with incoming information, which enables the maintenance and recall of the main ideas of a text (Cain et al. 2004; Daneman and Carpenter 1980; Stipek and Valentino 2015). Not surprisingly, WM appears to be an important predictor of RC in children aged from of 7 to 11 years after controlling other RC-related variables (Cain et al. 2004; Seigneuric et al. 2000), and WM deficits have been related to low RC performance in children (Cain et al. 2004; Nation et al. 1999; Seigneuric et al. 2000; Seigneuric and Ehrlich 2005; Vukovic and Siegel 2006; see Carretti et al. 2009 and Peng et al. 2018, for reviews).

## Working memory updating and reading comprehension

Working memory updating (WMU) is a crucial mechanism whereby information in WM that is no longer relevant is substituted by newer information (see Miyake et al. 2000; Morris and Jones 1990; Palladino et al. 2001). Updating plays a role in RC, allowing changes of mental text representations to be maintained in WM as the reading progresses. For example, information has to be updated when a new character appears, when the scenario in which the story takes place changes, or when new information comes into conflict with that provided so far. Therefore, WMU contributes to the integration of new and previous information, which constitutes a higher-level skill essential to RC (Cain and Oakhill 2004).

There is a wealth of empirical evidence showing the contribution of WMU to RC performance (Carretti et al. 2005; García-Madruga et al. 2014; Palladino et al. 2001; Pelegrina et al. 2015; Potocki et al. 2017; for meta-analyses, see Butterfuss 2017 and Follmer 2017; although see Artuso and Palladino 2016, for an absence of a relationship). WMU appears to be involved in RC, especially when deeper comprehension is necessary, as in the case of inferences (García-Madruga et al. 2014; Potocki et al. 2017).

An influential explanation of the relationship between WMU performance and RC postulates that poor comprehenders are less efficient at regulating the level of activation of information in WM (Carretti et al. 2005; Palladino et al. 2001). Thus, less-skilled comprehenders may experience difficulties in both WMU and RC tasks, to the extent that both tasks require regulating the level of activation of information and, specifically, inhibiting representations that are no longer relevant. Besides inhibitory function, other component processes are involved in updating. Indeed, updating is considered a complex process comprising several subprocesses components, such as retrieval or substitution of information (Bledowski et al. 2010; Ecker et al. 2010; Zhang et al. 2012). Investigating these components could provide a more detailed picture of the role of WMU in RC.

## WMU components

In the present study, we examined the contribution of the retrieval and substitution components to RC. These components have been shown to make independent contributions to performance on WMU tasks, and they are assumed to be combined serially because their contribution to reaction times is additive (Ecker et al. 2010). Thus, to update a representation held in WM, it first has to be retrieved and then, substituted by a new one. Both processes components are described below.

Retrieval refers to accessing information held in WM so that it can be selected for a cognitive operation. This is a time-consuming and sometimes error-prone process that involves searching for a specific representation among other candidate representations. To update an element, it has to be active in the focus of attention in WM which enables it to be immediately accessible for any cognitive operation (e.g., Cowan 2005; Oberauer 2002, 2009). Retrieval from WM is also essential during reading, because information must be constantly activated so that it can be updated. For instance, retrieval is often initiated by pronouns or anaphors (Garnham et al. 1992; Just and Carpenter 1992; McElree 2015). When a pronoun appears during reading, its antecedent must be accessed, sometimes by selecting it from several possible alternatives. In these situations, accessing the correct information is crucial to eventually update it and generate a coherent representation. To illustrate this, consider the following sentence: “Susan left her job as a shop assistant and joined a travel agency. She found it much more interesting and motivating.” In this example, “it” can refer to either of the two jobs in the previous sentence. Retrieving the appropriate antecedent (i.e., working in a travel agency) is crucial to better understand the continuation of the protagonist’s employment story. Similarly, during the reading of a text, information must be constantly updated, which often requires the continuous retrieval of information from WM. For instance, if the text above continues: “(…) After some time, Susan was tired of organizing trips and she got a position as marketing coordinator,” the reader must retrieve the previously updated information about the job of Susan.

Substitution is a secondary updating process that involves the replacement of a representation or subset of representations, while the rest of the information in WM is preserved and protected from interference. During reading, information changes rapidly and it becomes necessary to substitute contents that are no longer relevant. If the novel information is not integrated adequately within the text, part of the text may lose consistency such that it cannot be fully understood. Thus, a binding mechanism is also required to integrate the new information with that previously stored in a more complex representation (Artuso and Palladino 2011; Oberauer et al. 2017). Considering the above example, if the protagonist’s previous work was not replaced, the reader may get “stuck” in the story and not understand the motivations or events that may occur in this and other areas of the protagonist's life.

Some studies have found individual and age-related differences in specific components of the updating process. Unsworth and Engle (2008) observed that individuals with low WM capacity had more difficulty in retrieving information from outside of the focus of attention when performing a WMU task. Ecker et al. (2010) showed that retrieval accuracy in a WMU task was related to individual differences in WM capacity, whereas substitution accuracy was not. In a similar vein, Linares et al. (2016) observed age-related differences during childhood and adolescence in the ability to accurately retrieve information, although there were no differences in the ability to substitute information in an updating task. An issue that remains to be studied is whether individual differences in WMU components are also related to individual differences in RC.

## Present study

While a number of previous studies have shown the relationship between WMU and RC, they considered updating as a global process, which precludes evaluation of the separate roles of different component processes. The aim of the present study was to examine the specific contributions of retrieval and substitution WMU components to RC performance.

We used a set of numerical updating subtasks, each of which may or may not require the retrieval and substitution components. The separate effect of each updating component can be determined by comparing the performance on the different updating subtasks. In all of the subtasks, two numbers were associated with two boxes that served as contextual cues for two elements in WM. Also, all of the subtasks required the application of basic arithmetic operations to generate the numerical information used in the different trials. Depending on the components involved in each subtask, participants had to retrieve information for an element (cued by its box), perform various arithmetical operations and substitute the number for each box. For example, the task that entailed retrieval and substitution required the following processes for each item: *retrieving* the number associated with a box (e.g., 3); applying it an arithmetical operation (e.g., + 2) and *substituting* the previous number associated with that box by the obtained result (e.g., 5).

Recently, the relationship between RC and updating performance was shown to disappear when basic abilities, such as vocabulary or reading speed, were controlled for (Muijselaar and de Jong 2015). This suggests that the relationship between WMU and RC may depend on other skills that are necessary and common to both of them. Therefore, in addition to the experimental tasks, different abilities that could account for the relationship between WMU and RC performance were also assessed in this study. A fluid intelligence measure was included because previous research has shown that individual differences in fluid intelligence are related both to RC (e.g., Johann et al. 2019) and WMU (Belacchi et al. 2010; Chen and Li 2007; Cornoldi 2006; Friedman et al. 2006). Vocabulary was also controlled, given its relationship with both RC (e.g., Verhoeven and van Leeuwe 2008; Yovanoff et al. 2005) and WMU (Salthouse et al. 2003). Reading fluency and reading ability measures were also included, given that some studies have observed moderate-to-high positive correlations between measures of fluency and RC (Klauda and Guthrie 2008; Rasinski et al. 2005; Verhoeven and van Leeuwe 2008). Finally, a math task was administered because the updating tasks entailed arithmetical operations and also because there is evidence of a relationship with WMU (Bull and Lee 2014; Passolunghi and Pazzaglia 2004; Pelegrina et al. 2015) and reading skills (Lerkkanen et al. 2005).

In summary, the study was carried out with the objective of examining possible relations between retrieval and substitution WMU components and RC, after controlling for other RC-related variables. We are not aware of any previous research that has examined the separate influence of WMU components on RC. Given that the retrieval component is related to individual differences in WM capacity (Ecker et al. 2010) and undergoes age-related changes (Linares et al. 2016), we expected individual differences in RC to also be related to this component. We made no specific hypotheses regarding to the role of the substitution component, as several studies (Ecker et al. 2010; Linares et al. 2016; Unsworth and Engle 2008) failed to demonstrate specific involvement of this component in individual differences in complex abilities.

## Method

### Participants

A total of 162 children in the fourth grade (9–10 years old) took part in this study; they were all recruited from various local schools located in a neighborhood with middle socioeconomic status in a medium-sized city in southern Spain. From the initial sample, four children with neurological problems or special education needs were excluded and one dropped out during the final session. In addition, nine participants had to be excluded due to being given incorrect instructions by an experimenter. Thus, the final sample was composed of 148 children (64 boys and 84 girls; mean age: 9.39 years, *SD* = 0.49). All participants had Spanish as a first language. Written informed consent was provided by a parent or legal guardian, and verbal assent was obtained from the children prior to study commencement.

### Materials

#### Working memory updating

**Fig. 1:**
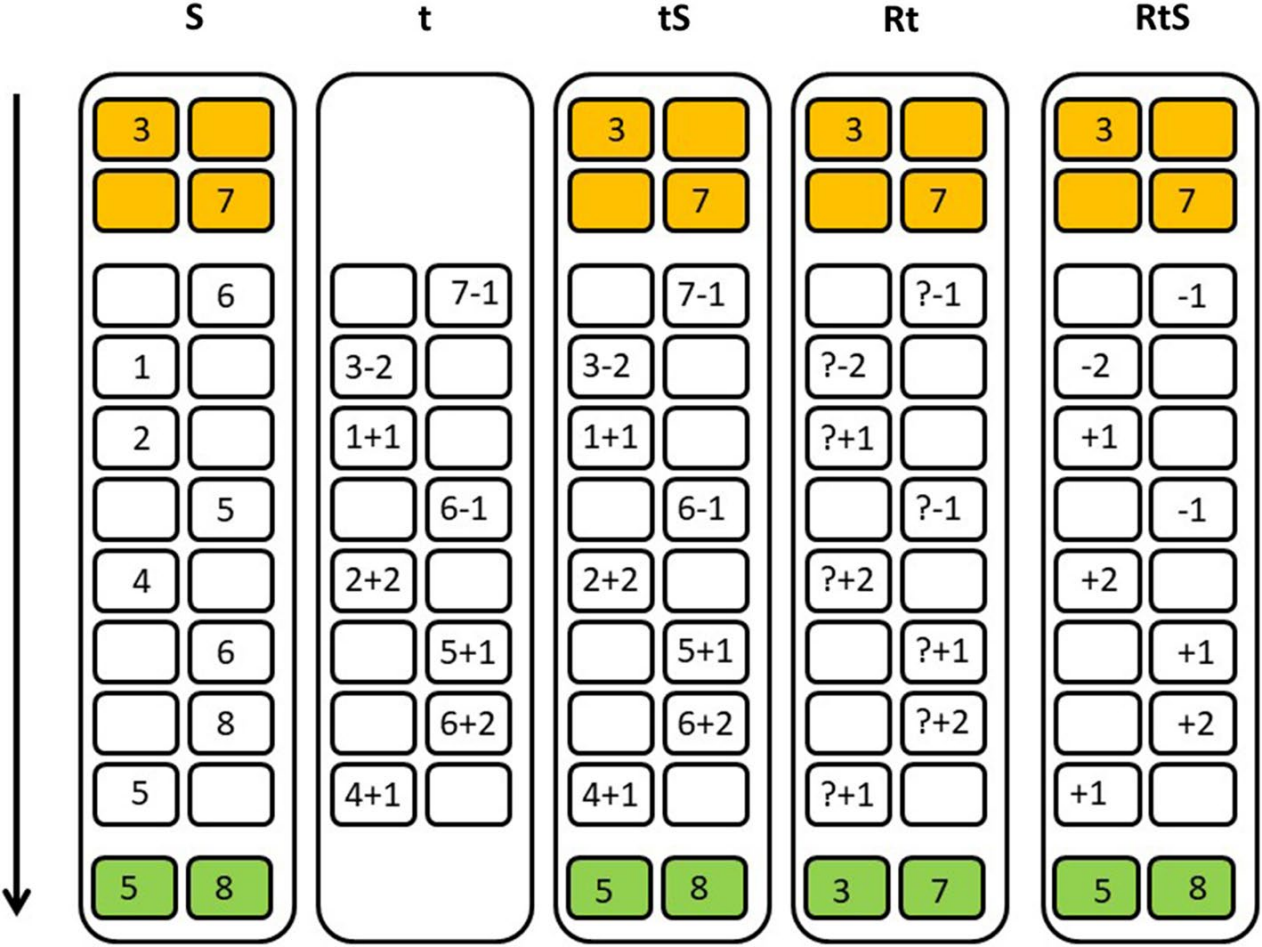
Representation of an example list for each subtask of the WMU task. *Note* Each rectangle represents a subtask, whose label is on the top. Within each rectangle, each column represents one of the two boxes presented simultaneously during the task and each row represents an item. Orange boxes indicate the initial items, white boxes the study items, and green boxes the recall items. In the S subtask, participants had to remember the last number presented for each box. In the t subtask, participants were required to solve simple arithmetical operations. In the tS subtask, participants had to solve arithmetic operations and memorize the last result obtained for each box. In the Rt subtask, the initial numbers had to be recalled; arithmetical operations were always applied to these numbers. For each item, participants had to type the result. In the RtS subtask, participants were to recall the number associated with each box, apply the arithmetical operation and type and memorize the result obtained in order to use it in the next operation

This task, adapted from Linares et al. (2016), included five numerical subtasks that required different updating components and were presented as various games. Figure 1 depicts an example of each subtask.

Each list started with the presentation of two initial numerical items (ranging from 1 to 6) within two rectangular boxes (one for each box: one on the right and one on the left, at the same height). Then, eight study items were displayed within one of the two rectangular boxes. Each item had a 50% probability of being associated with one of the boxes. Study items varied depending on the task and could be numbers, question marks, mathematical operations (− 1, + 1, − 2, + 2) or a combination thereof. Each list ended with the presentation of two empty boxes: participants had to type a number in each box that, depending on the subtask, could be the first two numbers of the list, or the last numbers or results. It should be noted that the current study focused only on the retrieval and substitution components. However, the transformation component, which entails the application of arithmetical operations, was also included (but not manipulated) as a way to obtain new numerical values in the different conditions that could be retrieved or substituted.

Each subtask was labeled with the first letter of the involved processes; thus, R indicated retrieval, S represented substitution and lowercase t (because it was not manipulated) denoted transformation. For example, the task RtS required retrieval, transformation and substitution. To make the distinction between subtasks easier for children, each task was presented as a game associated with a different picture (coffer, fishbowl, gift box, television, wicker basket and bag of money). These pictures were used as backgrounds, over which the boxes were superimposed.

The S subtask involved the substitution process. In this subtask, two initial numbers were displayed, one for each box, followed by eight additional numbers (e.g., 6, 4…). Participants had to memorize the initials numbers, and, when a new number appeared, they had to replace the old number of the same box with the newly presented number. Participants had to type the number displayed within each box to move on to the next item. At the end of the list, they were asked to type the last number memorized for each box. This subtask was associated with the picture of a fishbowl.

In the t subtask, only the transformation process was required; neither retrieval nor substitution were involved. In this subtask, no initial numbers were displayed. The list included eight items (consisting of mathematical operations) inside the boxes (e.g., 6 − 2, 4 + 1…). For each item, participants had to type the result of the operation to move on to the next item. Because no retrieval of information was required, there were no empty boxes at the end. This subtask was associated with the picture of a gift box.

The tS subtask involved transformation and substitution. This subtask started with the presentation of two initial numbers. Then, eight mathematical operations were displayed inside the boxes (e.g., 6 − 2, 4 + 1…). Participants had to type the result of each operation presented in a given box and remember it for possible recall. At the end of the list, participants had to type the last result associated with each box. This subtask was associated with the picture of a television.

The Rt subtask required retrieval and transformation. In this subtask, after the two initial numbers, eight items, i.e., a question mark and one arithmetical operation (e.g., ?− 2, ? + 1…), were displayed. Participants had to remember the initial numbers and apply each operation to the initial number for the same box and type the result. At the end of the list, participants were asked to type the two numbers memorized initially. Because no substitution process was required, the numbers maintained in memory did not change throughout the trials. This subtask was associated with a picture of a wicker basket.

The RtS subtask involved all three processes: retrieval, transformation and substitution. Two initial numbers had to be memorized. Then, eight items consisting of arithmetical operations were displayed inside of one of the two boxes (e.g., − 2, + 1…). Participants had to retrieve the number for the actual box, apply the operation and type and memorize the new result for use in subsequent items. At the end of the list, two empty boxes appeared and participants had to type the last result obtained for each box. This subtask was associated with the picture of a bag of money.

The complete sequence of tasks was divided into two blocks. In the first block, the five subtasks were arranged as follows: S, t, tS, Rt and RtS. Presenting the simpler tasks at the beginning made it easier for the children to understand the procedures for the more complex ones. In the second block, the subtasks were presented in the opposite order: RtS, Rt, tS, t and S. Reversing the order of the tasks in the second block equalized the presentation order of the tasks. By averaging the serial order of the tasks, the practice effects and fatigue effects were more balanced. For each subtask, eight lists were constructed, so that four lists of each subtask were presented in each block. The whole task involved 40 experimental and 20 practice trials.

#### Reading comprehension

RC was assessed with a reading comprehension test (ECOMPLEC; León et al. 2012). In this task, the children had to read two texts (one narrative and one expositive) and answer some multiple-choice questions about them. The first text, entitled “El hombrecito sabelotodo” (The all-knowing little man), was a narrative text of 542 words, while the second text, “Los glóbulos rojos” (The red blood cells), was an expository text of 348 words (five paragraphs) that describes the functions and characteristics of the circulatory system. There were 22 questions about the first text and 21 about the second one. The participant’s score corresponded to the total number of correct responses (range: 0–43). The test time was 40 min. Cronbach’s alpha, as reported in the manual, is 0.89.

#### Fluid intelligence

The Culture Fair test, scale 2 (Cattell and Cattell 1973), consists of two parallel forms (A and B), each containing four subtests. In the present study, two subtests were administered: series and classification. In the series subtest, the children were presented with an incomplete series of abstract figures and had to select the best option (among five) to complete the series. In the classification subtest, the children were presented with a series of problems consisting of abstract figures and had to determine the odd figure in a set by selecting the best option among various possible solutions. The administration time of the two scales was 7 min. The split-half reliability reported in the manual is 0.84.

#### Math fluency

The Spanish version of the Woodcock-Johnson III (WJ-III) Math Fluency subtest (Muñoz-Sandoval et al. 2005) was used. The children had to perform as many simple arithmetical operations (addition, subtraction and multiplication) as they could within 3 min. The maximum number of correct operations was 160. The test–retest reliability coefficient reported in the manual for 9-year-olds is 0.95.

#### Reading fluency

The Spanish version of the Woodcock-Johnson III (WJ-III) reading fluency subtest (Muñoz-Sandoval et al. 2005) was used; the children had to read and judge the veracity of, as many sentences as they could within 3 min. The maximum score was 105. The test–retest reliability coefficient reported in the manual for 9-year-old children is 0.96.

#### Vocabulary

The Vocabulary scale of PMA battery (Thurstone and Thurstone 2002) was used; the children had to select the best synonym for a given target word among four possible solutions, as many items as they could within 4 min. The maximum score was 50. The split-half reliability reported in the manual is 0.91.

#### Reading ability

The Word Reading subtest of the LEE battery (Defior et al. 2006) was used; the children had to read a list of 42 words as quickly as possible, without errors. The time (in seconds) spent reading the list served as the dependent variable. The test–retest reliability coefficient reported in the manual is 0.88.

### Procedure

This study was conducted over two sessions that took place within school classrooms. In the first session, children were tested in the classroom during an hour of class time. All children completed the paper and pencil tasks in the following order: RC, fluid intelligence (Cattell), math and reading fluency (WJ-III) and vocabulary (PMA). Between tasks, the experimenter provided reinforcement regarding the work that the children were doing to keep them motivated. In addition, the brevity of the tasks made the session more dynamic, which helped maintain the children’s interest.

In the second session, the children were assessed individually in a quiet room within their school for an hour. In this session, the Word Reading task and computerized WMU tasks were administered. The WMU tasks were computer-administered using a laptop with a 12.1-inch screen. Instructions were given before starting each WMU subtask. Once the experimenter was confident that the participant had understood the subtask, and after two practice trials had been completed, the subtask began. The same procedure was repeated for each subtask. The Word Reading task was administered once the participant had finished the WMU tasks.

### Data analysis

Linear mixed models were used to determine the relationships between RC and the WMU components: retrieval and substitution. In these models, time and accuracy in the updating tasks were used as dependent variables, and WMU components, RC and the rest of individual variables were entered as predictors. The specific question of interest was whether RC or its interaction with substitution and retrieval would account for individual differences in updating (time and accuracy) after controlling for differences in other cognitive variables.

Initially, the random-effect structure was determined by including a random intercept for participants. The variables were then entered as follows: First, as fixed effects, WMU components (retrieval, substitution) and their interaction term were entered. Second, all control variables (Cattell, math fluency, reading fluency, vocabulary and reading ability) were included, and third, RC and all two- and three-way interaction terms, including RC, were entered. In a step-wise procedure, non-significant interactions and predictors were excluded. In each step, a larger model was iteratively compared with the previous one using the log-likelihood ratio test, until the simplest model with adequate fit was obtained. Thus, the final model contained a reduced number of predictors and interaction terms than the most complex model. Separate analyses were performed for response times and accuracy. This analysis was carried out using linear mixed-effects models with the lme4 package (Bates et al. 2014) in the R environment (R Core Team 2020).

## Results

Response times for the practice lists were excluded. Response time data for lists with incorrect recall were discarded from the analyses (11.19% of all data). Response times lower than 200 ms, and those exceeding the participant’s mean by more than 3.5 standard deviations, were also removed (2.04% of data). Accuracy was calculated as the percentage of correct answers obtained on the entire list. Means, standard deviations and correlations among the variables considered in this study are shown in Table 1.

**Table 1:** Descriptive statistics and zero-order correlations between response times and errors on each measure

	Mean	*SD*	Time (S)	Time (t)	Time (TS)	Time (Rt)	Time (RtS)	Acc (S)	Acc (t)	Acc (tS)	Acc (Rt)	Acc (RtS)	RC	Cattell	MF	RF	Voc	RA
Time (S)	2002	639	–															
Time (T)	2617	866	.32**	–														
Time (TS)	3789	1350	.62**	.79**	–													
Time (RT)	4428	1374	.39**	.66**	.69**	–												
Time (RTS)	4095	1553	.50**	.64**	.74**	.60**	–											
Acc (S)	98.69	2.13	− .31**	− .24*	− .31**	− .28**	− .20*	–										
Acc (T)	97.00	3.18	.11	− .23*	− .13	− .12	− .13	.21*	–									
Acc (TS)	96.81	3.51	.00	− .35**	− .21*	− .25*	− .28**	.17*	.33**	–								
Acc (RT)	84.08	13.77	.10	− .19*	− .03	− .25*	− .06	.10	.28**	.52**	–							
Acc (RTS)	70.05	18.39	.09	− .25*	− .09	− .28**	− .31**	.07	.12	.45**	.52**	–						
RC	26.89	6.24	− .15	− .20*	− .23*	− .18*	− .25*	.06	.07	.09	.17*	.23*	–					
Cattell	13.18	3.40	− .15	− .38**	− .30**	− .35**	− .37**	.12	.21*	.30**	.36**	.41**	.39**	–				
MF	60.16	18.79	− .22*	− .71**	− .58**	− .45**	− .46**	.18*	.26**	.37**	.16	.30**	.29**	.36**	–			
RF	45.66	10.80	− .20*	− .39**	− .34**	− .29**	− .31**	.03	.13	.17*	.17*	.25*	.49**	.41**	.42**	–		
Voc	13.93	5.23	− .15	− .29**	− .26*	− .17*	− .25*	− .06	.02	− .02	.01	.09	.43**	.36**	.28**	.64**	–	
RA	41.61	12.03	.11	.39**	.28**	.14	.25*	.01	− .20*	− .15	− .13	− .21*	− .25*	− .30**	− .36**	− .39**	− .33**	–

* *p* < .05, ** *p* < .001

*RC* reading comprehension, *RF* reading fluency, *MF *math fluency, *Voc* vocabulary, *RA* reading ability and *Acc* accuracy

Response times are given in milliseconds and accuracy as percentage of correct recall

### Response times

First, we examined whether response times were related to retrieval and substitution. In the model that included retrieval and substitution as predictors, response times increased when information had to be retrieved or substituted. The interaction between the two updating components revealed that the substitution effect differed depending on the involvement of the retrieval component. To decompose the interaction, a model including substitution as a predictor was run for each level of retrieval. The separate analyses showed a significant temporal cost of substituting information when retrieval was not required (*Mdiff* = 278 ms). In contrast, when retrieval was necessary, response times were longer when substitution was not required than when it was required (*Mdiff* = 333 ms). This result replicates the findings reported in previous studies using other version of the tasks (Linares et al. 2016; Pelegrina et al. 2020).

In the following steps, control variables potentially related to WMU and RC were successively added to the model. Only fluid intelligence and math fluency scores accounted for the additional variance, with response times decreasing with increasing scores on both variables.

Finally, the main variable of interest, RC, as well as its interactions with retrieval and substitution, was successively added to the model. Neither RC nor its interaction terms explained additional variance in response time. Therefore, RC was not related to the time spent substituting or retrieving information in the WMU tasks. Table 2 provides the parameter estimates for the final model in terms of the response times. Model fit statistics are provided in Supplementary Table S1, and the results of model comparisons are presented in Supplementary Table S2.

**Table 2:** Estimates of fixed effects for response times and accuracy

Dependent variable	Effect	Estimate (SE)	*df*	*t*	*p*	*95% CI*
Time	Intercept	4989.03 (289.60)	169.51	17.22	< .001	[4421.41, 5556.65]
	Retr	1811.13 (117.80)	592.00	15.37	< .001	[1580.24, 2042.02]
	Subst	278.27 (102.02)	592.00	2.72	0.006	[78.31, 478.23]
	Retr x Subst	− 611.02 (155.84)	592.00	− 3.92	< .001	[− 916.47, − 305.57]
	Subst in Retr	− 332.8(107.9)	148.00	− 3.08	0.002	[− 544.28, − 121.32]
	Subst in No-Retr	278.3(112.3)	296.00	2.47	0.013	[58.19, 498.41]
	Cattell	− 57.46 (19.84)	148.00	− 2.89	0.004	[− 96.35, − 18.57]
	Math fluency	− 26.84 (3.59)	148.00	− 7.46	< .001	[− 33.88, − 19.80]
Accuracy	Intercept	88.84(2.69)	265.20	33.00	< .001	[83.57, 94.11]
	Retr	− 26.22 (3.20)	592.00	− 8.19	< .001	[− 32.49, − 19.95]
	Subst	0.74 (0.93)	592.00	0.80	0.422	[− 1.08, 2.56]
	Reading Comp	− 0.10 (0.09)	243.54	− 1.11	0.266	[− 0.28, 0.08]
	Retr x Subst	− 14.32 (1.42)	592.00	− 10.03	< .001	[− 17.10, − 11.54]
	Subst in Retr	− 13.57(1.33)	148.00	− 10.15	< .001	[− 16.18, − 10.96]
	Subst in No-Retr	0.74(0.27)	296.00	2.68	0.007	[0.21, 1.27]
	Retr x Reading Comp	0.49 (0.11)	592.00	4.41	< .001	[0.27, 0.71]
	Cattell	0.77 (0.14)	148.00	5.16	< .001	[0.50, 1.04]
	Math fluency	0.06 (0.02)	148.00	2.63	0.009	[0.02, 0.10]
	Vocabulary	− 0.23 (0.09)	148.00	− 2.45	0.015	[− 0.41, − 0.05]

### Accuracy

In a second set of analyses, we determined whether the updating components had an effect on accuracy. The initial model, including retrieval and substitution as predictors, revealed that accuracy decreased when information had to be retrieved. Furthermore, the interaction between both updating components was significant. To further analyze this interaction, a model including substitution as a predictor was run for each level of retrieval. These analyses showed that substitution significantly decreased accuracy when retrieval was required (*Mdiff* = 14%). However, when retrieval was unnecessary, substitution in fact induced a small increase in accuracy (*Mdiff* = 0.75%).

In the following steps, control variables related to WMU were successively added to the model. Only fluid intelligence, math fluency and vocabulary scores significantly predicted accuracy. Specifically, accuracy increased with fluid intelligence and math fluency and decreased with vocabulary scores (Table 2 shows the parameter estimates for the final model with respect to accuracy). The unexpectedly negative effect of vocabulary may reflect statistical suppression, which can occur when several correlated control variables are included in a model (see Tzelgov and Henik 1991). It should be noted that vocabulary had negligible correlations with all the accuracy measures (see Table 1). Further examination of the beta values showed that vocabulary also failed to reach significance when Cattell was excluded from the model (*b* = − 0.14, *p* = 0.16); however, when both vocabulary and Cattell were included as predictors in the model, vocabulary had a significant negative effect (*b* = − 0.23; *p* < 0.05) and the coefficient (*b*) for Cattell increased from 0.70 (*p* < 0.001) to 0.77 (*p* < 0.001). Thus, vocabulary suppressed irrelevant variance in Cattell and became a negative predictor of accuracy. Importantly, despite this suppressor effect, vocabulary did not influence the effects under study; the effects of recall, substitution, comprehension and their interactions did not differ substantially between models including and omitting vocabulary as a predictor.

**Fig. 2:**
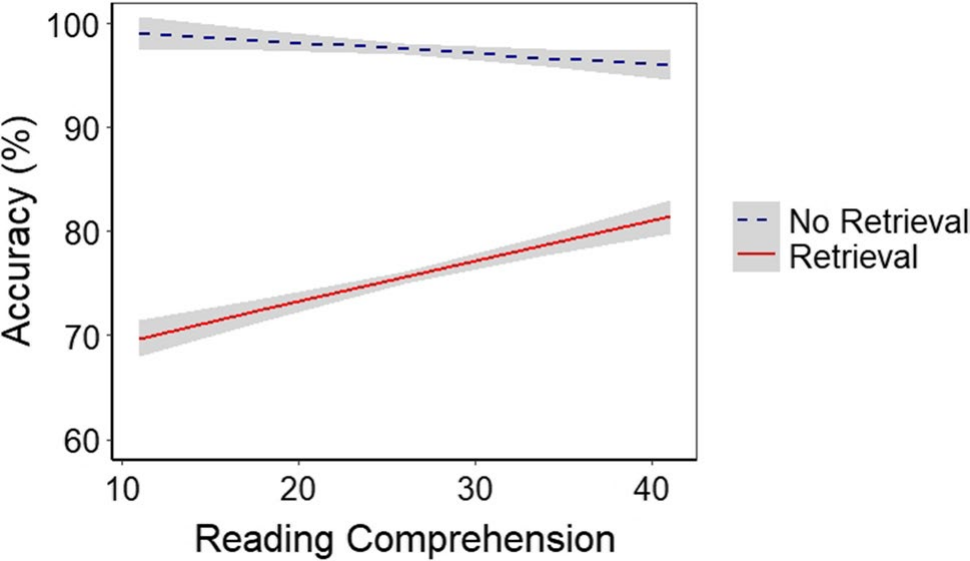
Reading comprehension effect on updating accuracy. *Note* Regression fit lines for the relationship between RC and updating accuracy. Lines are shown in blue and red for no retrieval and retrieval conditions, respectively. The gray shaded area represents standard error

In the final steps, RC and its interactions with retrieval and substitution were successively added to the model. The final model included RC and its interaction with retrieval. This interaction indicated that, when retrieval was required, accuracy increased as RC scores increased (see Fig. 2). Table 2 presents the parameter estimates for the final model in terms of accuracy. Model fit statistics are provided in Supplementary Table S1, and the results of the model comparisons are presented in Supplementary Table S2.

## Discussion

The present study examined the relationships of retrieval and substitution WMU components with RC performance. We administered a WMU task comprising various subtasks that allowed the effects of the individual WMU components to be isolated. RC was also assessed, as well as performance on several RC-related tasks (fluid intelligence, reading and math fluency, vocabulary and reading ability) that were included to control for basic and general cognitive abilities (see Cain and Oakhill 2006; Cornoldi and Oakhill 1996). The results indicated that, among WMU components, retrieval was uniquely related to individual differences on RC performance, where this relationship held even after controlling for general abilities related to reading.

### WMU components

Updating tasks require constant retrieval of information from WM, which frequently must be used in combination with new information. The retrieval component had a considerable effect on WMU performance, as longer response times and lower accuracy were observed when retrieval was required than when it was not necessary. A representation to be processed in WM must be in an active and highly accessible state, i.e., must be the focus of attention. The capacity of the focus of attention is extremely limited such that, under normal conditions, only one element can be processed immediately (McElree 2001; McElree and Dosher 2001; Oberauer 2002). A reduced number of additional elements may also be maintained in WM in a less accessible state, such that they must be retrieved into the focus of attention every time; they have to be updated or processed. Even in an updating task with only two elements, as used in this study, time and accuracy costs are evident when information has to be retrieved into the focus of attention (see Garavan 1998). It has been proposed that representations in WM are maintained and accessed in the same way during language comprehension and WM tasks (see McElree 2015, for a review). McElree et al. (2003) provided compelling evidence that retrieval in WM is based on a direct-access operation rather than an element-by-element search. Evidence of direct access to information outside the focus of attention has been found for both written comprehension (McElree 2000; McElree et al. 2003) and oral comprehension (Johns et al. 2015). On this basis, we assume that this direct-access mechanism underlies information retrieval during both comprehension and WMU tasks.

Importantly, we found a relationship between the retrieval of information and RC performance. Specifically, participants who showed lower RC performance also exhibited lower updating accuracy when information had to be retrieved into the focus of attention. However, no such relationship was observed with respect to the time needed to retrieve information. This finding is germane to the distinction between availability and accessibility (e.g., Vaughan et al. 2008). Accessibility of information in WM refers to how fast a representation is activated in the focus of attention, whereas availability indicates the accuracy of retrieval of information for further processing. Thus, although less-skilled comprehenders can access the information as efficiently as good comprehenders, their representations outside the focus of attention are less available. This result is consistent with those obtained by Johns et al. (2015) using an auditory comprehension task based on the procedure developed by McElree et al. (2000, 2003). They demonstrated that less-skilled readers showed lower accuracy but equal retrieval speed compared to skilled readers in an auditory task. Therefore, low retrieval accuracy seems to be a characteristic of poor readers in both language and WMU tasks.

There are two plausible explanations (which are not mutually exclusive) for the decrease in availability of information outside the focus of attention for less-skilled comprehenders: difficulties in using retrieval cues and lower representational quality. With regard to the first explanation, poor comprehenders might fail to access the correct information cued by the context more frequently. In the present WMU task, the context represented by the box could not activate the associated representation, thereby hindering the subsequent recall of information. Good access to cued information is also crucial in RC. For instance, when a pronoun appears during reading, access and selection of its correct antecedent, especially if there is more than one possible antecedent, will determine the degree of comprehension of the text. Previous studies have shown that pronoun resolution is related to RC (e.g., Oakhill and Yuill 1986; Eilers et al. 2019). Thus, when the pronoun “she” appears in a story with two female characters, correct pronoun resolution and subsequent retrieval of the name of the appropriate protagonist will promote comprehension. On the contrary, if the reader ultimately attributes the action to the wrong character, poorer overall comprehension is likely. Additional support for this explanation comes from recent evidence, showing that RC is related to recollection in a WMU recognition task (Pelegrina et al. 2023). Recollection involves the retrieval of information accompanied by contextual details. Thus, during reading, some information may be incorrectly recognized if its contextual cues are not correctly retrieved.

In addition to difficulties in using retrieval cues, less-skilled comprehenders might retrieve information less accurately because their reading-related memory representations are qualitatively diminished. Representations outside the focus of attention suffer from decay or interference, or both, which lowers their quality (e.g., McElree 2001; Vaughan et al. 2008). In fact, a higher susceptibility to interference has been considered as a major cause of the difficulties experienced by poor comprehenders. A number of studies have shown that less-skilled comprehenders show more recall intrusions in WMU tasks (e.g., Borella et al. 2010; Carretti et al. 2005, Palladino et al. 2001; Pelegrina et al. 2015). Although these difficulties have been attributed to inhibitory deficits, they may also be due, at least in part, to interference between representations held in WM that would ultimately result in a reduction in the quality of the representations.

The different retrieval requirements of updating tasks may explain why some recent studies failed to observe a relationship between WMU performance and RC (Muijselaar and de Jong 2015; Artuso and Palladino 2016). One reason for the inconsistency with previous research is that these latter studies (e.g., Artuso and Palladino 2016) included WMU tasks that depended mainly on the substitution component and did not require the retrieval of information. In other studies, using a running memory task, performance was suggested to rely on recency effects (Muijselaar and de Jong 2015). In this task, participants may passively process items (see Elosúa and Ruiz 2008), so retrieval would play a minor role. In contrast, studies in which updating has been consistently related to RC made use of tasks in which retrieval is necessary. For instance, in the semantic updating task (Carretti, et al. 2005; Palladino et al. 2001; Pelegrina et al. 2015; Sorqvist et al. 2010), new information and information held in memory must be compared, which requires the latter to be retrieved. Therefore, a tentative explanation for some of the inconsistent results is that updating performance may contribute to RC when information in the WMU task has to be retrieved.

Substitution is the component that could most readily be associated with updating, because it is present in all WMU tasks (Ecker et al. 2010). In the current study, the requirement for substitution had an effect on performance; however, neither the accuracy nor speed with which information was substituted was related to RC performance. This indicates that even less-skilled comprehenders can perform substitution adequately, in line with other studies that did not observe individual or age differences in substitution performance during updating tasks (Ecker et. al., 2010; Linares et al. 2016). Recently, Frischkorn et al. (2022) showed that substitution made a relatively modest contribution (15%) to individual differences in performance in updating tasks; moreover, this component was not related to higher cognitive abilities, such as reasoning, nor to WM measures. Therefore, although substitution is an essential component of updating, it does not seem to be a critical determinant of individual differences in various domains, including RC.

It is worth to note that the WMU tasks included numbers rather than words as stimuli. Therefore, the present data suggest that RC difficulties are related to general updating difficulties. A recent meta-analysis (Peng et al. 2018) shows that reading performance is significantly related to WM, regardless of the material used, although this relationship is stronger for verbal than for numerical material and the latter with respect to visuospatial material. Using updating tasks, Pelegrina et al. (2015) found that children with RC difficulties performed worse when the task included word stimuli than in an analogous task with numerical material. Therefore, the nature of the material may modulate the difficulties in updating information associated with poor RC. It would be informative to compare performance in this WMU paradigm using different types of information (i.e., words, number and visuospatial material).

RC is a complex task which entails a number of cognitive processes, including making inferences, or substitution of mental model elements that require updating information. Further research is needed to determine the role of the different updating component in these processes. In addition, it would be interesting to explore whether training the retrieval WMU component leads to improvements in RC. One way to do this would be to embed the retrieval tasks in RC activities. This approach to WM training has been shown to improve children’s reading comprehension (García-Madruga et al. 2013; Carretti et al. 2017).

In conclusion, this study, which focused on the role of two components of updating, provides evidence that retrieval is a WMU component linked to individual differences in RC. In particular, poorer RC performance is related to greater difficulties in retrieving information accurately. Retrieval difficulties in WMU could in part explain the relation between RC and WMU performance found in previous studies (for reviews, see Butterfuss and Kendeou 2017 and Follmer 2017). In contrast, substitution does not account for individual differences in RC, even though it is an essential component in updating tasks.

## Supplementary Information

Below is the link to the electronic supplementary material.

Supplementary file1 (PDF 227 kb)
