# Supplementary material for: The relationship between working memory updating components and reading comprehension
Source: Cogn Process. 2023 Feb 11;24(2):253–65. doi: 10.1007/s10339-023-01127-3 (PMC10110648; doi:10.1007/s10339-023-01127-3)
Supplement: Supplementary file 1 — Supplementary file1 (PDF 227 kb) [file 10339_2023_1127_MOESM1_ESM.pdf]

Cognitive Processing

The relationship between working memory updating components and reading comprehension

Rocío Linares and Santiago Pelegrina

Department of Psychology, University of Jaén, Spain

Author Note

Correspondence should be addressed to Rocio Linares, Universidad de Jaén, Paraje Las Lagunillas s/n, 23071 Jaén (Spain), Email: rlinares@ujaen.es

Supplementary Table S1

Model fit statistics for response times and accuracy

	Model	Fixed effects	AIC	BIC	logLik	df
Time	1	Retr	12592	12611	-6292.1	4
	2	Retr + Subst	12594	12617	-6292.1	5
	3	Retr + Subst + Retr x Subst	12581	12609	-6284.5	6
	4	Retr + Subst + Retr x Subst + Ct	12559	12591	-6272.5	7
	5	Retr + Subst + Retr x Subst + Ct + Fm	12514	12551	-6248.9	8
	6	Retr + Subst + Retr x Subst + Ct + Fm + Rf	12514	12556	-6248.0	9
	7	Retr + Subst + Retr x Subst + Ct + Fm + Voc	12515	12556	-6248.5	9
	8	Retr + Subst + Retr x Subst + Ct + Fm + Ra	12515	12557	-6248.7	9
	9	RC + Retr + Subst + Retr x Subst + Retr x RC + Ct + Fm	12515	12561	-6247.4	10
	10	RC + Retr + Subst + Retr x Subst + Subs x RC + Ct + Fm	12517	12563	-6248.5	10
	11	RC + Retr + Subst + Retr x Subst + Retr x Subs x RC + Ct + Fm	12517	12572	-6246.6	12
Accuracy	1	Retr	5683.8	5702.3	-2837.9	4
	2	Retr + Subst	5639.3	5662.4	-2814.7	5
	3	Retr + Subst + Retr x Subst	5551.1	5578.1	-2769.6	6
	4	Retr + Subst + Retr x Subst + Ct	5518.4	5550.6	-2752.2	7
	5	Retr + Subst + Retr x Subst + Ct + Fm	5514.8	5551.6	-2749.4	8
	6	Retr + Subst + Retr x Subst + Ct + Fm + Rf	5516.7	5558.2	-2749.3	9
	7	Retr + Subst + Retr x Subst + Ct + Fm + Voc	5512.0	5553.5	-2747.0	9
	8	Retr + Subst + Retr x Subst + Ct + Fm + Voc + Ra	5512.9	5558.9	-2746.4	10
	9	RC + Retr + Subst + Retr x Subst + Retr x RC + Ct + Fm + Voc	5495.5	5546.2	-2736.8	11
	10	RC + Retr + Subst + Retr x Subst + Subs x RC + Ct + Fm + Voc	5496.0	5551.3	-2736.0	12
	11	RC + Retr + Subst + Retr x Subst + Retr x Subs x RC + Ct + Fm + Voc + Ra	5496.0	5555.9	-2735.0	13

Supplementary Table S2

Results of model comparisons for response times and accuracy

	Model	χ^2	*df*	*p*
Time	Model 1 vs. Model 2	0.04	1	.833
	Model 1 vs. Model 3	15.22	2	< .001
	Model 3 vs. Model 4	24.00	1	< .001
	Model 4 vs. Model 5	47.34	1	< .001
	Model 5 vs. Model 6	1.68	1	.194
	Model 5 vs. Model 7	0.72	1	.395
	Model 5 vs. Model 8	0.32	1	.567
	Model 5 vs. Model 9	2.89	2	.235
	Model 5 vs. Model 10	0.75	2	.685
	Model 5 vs. Model 11	4.52	4	.340
Accuracy	Model 1 vs. Model 2	46.49	1	< .001
	Model 1 vs. Model 3	90.20	1	< .001
	Model 3 vs. Model 4	34.76	1	< .001
	Model 4 vs. Model 5	5.59	1	.018
	Model 4 vs. Model 6	0.07	1	.784
	Model 6 vs. Model 7	4.78	1	.028
	Model 7 vs. Model 8	1.11	1	.291
	Model 7 vs. Model 9	20.46	2	< .001
	Model 9 vs. Model 10	1.50	1	.219
	Model 9 vs. Model 11	3.5	2	.173
